# Use of hospitalisation history (lookback) to determine prevalence of chronic diseases: impact on modelling of risk factors for haemorrhage in pregnancy

**DOI:** 10.1186/1471-2288-11-68

**Published:** 2011-05-17

**Authors:** Jian Sheng Chen, Christine L Roberts, Judy M Simpson, Jane B Ford

**Affiliations:** 1 Clinical and Population Perinatal Health Research, Kolling Institute of Medical Research, Sydney Medical School, University of Sydney, Sydney, Australia; 2 Sydney School of Public Health, University of Sydney, Sydney, Australia

## Abstract

**Background:**

Concern about the completeness of comorbidity information in hospital records has been raised as a limitation of using hospital discharge data for research. The aim of this study is to assess the impact of additional comorbidity information from prior hospital admissions on estimation of prevalence and modelling of risk factors for obstetric haemorrhage.

**Methods:**

A range of chronic disease prevalence for 53,438 women who had their first birth in New South Wales (NSW), Australia, 2005-2006, were ascertained for up to five years prior to the birth admission (for pregnancy, 2-, 3-, 4- and 5-year periods) and obstetric haemorrhage was identified from maternal hospital records for 2005 and 2006.

**Results:**

The ascertainment of chronic disease prevalence increased with increasing length of lookback. However, the rate of the increase was slower after 2 to 3 years than for the more recent periods. The effect size of chronic diseases on obstetric haemorrhage risk decreased with the increased case ascertainment associated with longer lookback. Furthermore, longer lookback did not improve the predictive capacity (C-statistic: 0.624) of a model that was based only on the birth admission records.

**Conclusions:**

Longer ascertainment periods resulted in improved identification of chronic disease history among pregnant women, but the additional information from prior admissions did little to improve the modelling of risk factors for obstetric haemorrhage.

## Background

The use of population health data for health and health outcomes research is increasing. These routinely collected data may be administrative, surveillance, registry or vital statistics collections and have the common feature of including information on an entire population. However, concerns about the completeness of comorbidity information in the admission of interest (index record) have been raised as a limitation of using hospital discharge data for research [1]. One reason that comorbidity information is under-ascertained from hospital records is that only diagnoses affecting the current admission are required to be coded in the discharge summary, so unrelated chronic illnesses may not be recorded [2]. However, through record linkage it is possible to evaluate a patient's hospitalisation history in detail. Records belonging to the same individual can increasingly be longitudinally linked. The term that refers to identifying disease prevalence from health records that precede the record or event of interest is 'lookback' [3].

Using a longer lookback period for ascertaining a condition is likely to result in a higher proportion of subjects with the condition, but the effect of the condition may be reduced because the severity of the condition can vary depending on how recently it was identified [4]. Few studies have assessed the impacts of different lookback periods on ascertaining comorbidities, and almost all focused on the predictive performance of a comorbidity score in modelling of in-hospital or post-hospital mortality or readmission [3,5-8]. Little is known about the most appropriate lookback period for ascertaining comorbidities with regard to disease prevalence and risk estimation, predictive ability and statistical modelling of other outcomes. This is especially true in pregnancy which usually occurs among women who are relatively young and healthy. In Australia, 14% of female hospitalizations are related to pregnancy and childbirth. To date, lookback studies have been limited to older populations and the utility of the approach in pregnancy is unknown.

Worldwide, obstetric haemorrhage is a leading cause of maternal mortality and accounts for about 25% of all maternal deaths [9]. Increased rates of haemorrhage following childbirth have been observed in recent years in Australia, Canada, USA and Scotland [10]. Risk factors for obstetric haemorrhage include chronic diseases, advanced maternal age, obesity, cesarean section, multiple births, and induction and augmentation of labor [11-13]. Obstetric haemorrhage is therefore a suitable outcome to use for examining the effect of different lookback periods on ascertainment of risk factors and their prediction of subsequent outcome. In this study, we used longitudinally linked hospital discharge records to (1) assess impacts of different lookback periods on ascertainment of chronic disease history in pregnant women and (2) examine effects of increased ascertainment on modelling of risk factors for obstetric haemorrhage.

## Methods

### Study population and data sources

In the State of New South Wales (NSW), Australia, comprehensively linked perinatal population data were available from 1 July, 2000 to 31 December, 2006. Details of the record linkage were reported in a previous study [14]. For the current study we selected a population of pregnant women with five years of lookback and focused on women in their first pregnancy. Women with a previous pregnancy would have prior maternal admissions and might therefore have more opportunities for identification of chronic diseases in hospital data than women without a previous pregnancy. Study subjects included 55,002 women who had their first birth in NSW during 1 July, 2005 to 31 December, 2006. These women were identified from the NSW Midwives Data Collection ('birth data'). The birth data contain information on all births in NSW, including number of previous births, maternal health (including pre-existing hypertension), pregnancy, labour, delivery and perinatal outcomes. The birth data include information on live births or stillbirths of at least 20 weeks gestation or at least 400 grams birth weight.

**Figure 1:**
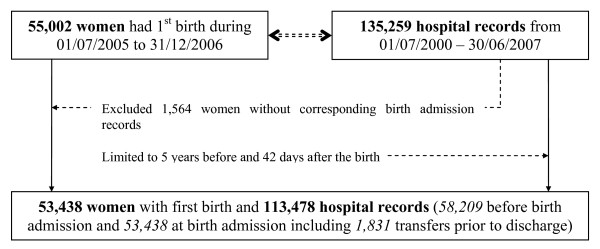
**Selection procedures for the study women and their hospital records**.

The NSW Admitted Patient Data Collection ('hospital data') covers every inpatient admission in NSW, and includes demographic and episode-related data. Data from the medical records are coded according to the tenth revision of the International Classification of Diseases Australian Modification (ICD-10-AM) and the affiliated Australian Classification of Health Interventions [15]. Up to 20 diagnoses and 20 procedures were used for disease identification in this study. Figure 1 presents the selection procedure of study subjects and hospital records. This study was approved by the NSW Population and Health Services Research Ethics Committee.

### Ascertainment of diseases

We selected chronic diseases including cardiac diseases, chronic renal disease, asthma/chronic obstructive pulmonary disease (COPD), psychiatric disorders, pre-existing hypertension, pre-existing diabetes, thyroid disorders and autoimmune diseases, for the study. The autoimmune diseases include Crohn's disease, ulcerative colitis, lupus, idiopathic, thrombocytopenic purpura, multiple sclerosis, psoriasis, autoimmune thyroiditis, rheumatoid diseases, Coeliac disease, vasculitis, pernicious anemia, myasthenia gravis, autoimmune hepatitis, ankylosing spondylitis, polymyositis and primary biliary cirrhosis. There is some evidence suggesting increased risk of obstetric haemorrhage associated with cardiac disease, pre-existing hypertension, asthma and thyroid disorders [11,16-18]. Based on biological plausibility, and since it is not clear that others have investigated potential associations with obstetric haemorrhage, renal disease, psychiatric disorders, diabetes and autoimmune diseases were also included as potential risk factors for haemorrhage. Given that there are relatively few population-based studies of risk factors for obstetric haemorrhage and that the chronic diseases are relatively rare among pregnant women, our large sample of pregnancies represented an ideal opportunity to investigate potential influence of other chronic diseases on haemorrhage risk. These diseases were chosen because their chronic nature means that they would still be present at the time of the birth regardless of the lookback period chosen.

The ICD-10-AM diagnosis and affiliated procedure codes for these chronic diseases are presented in Table 1 in the Appendix. Cardiac diseases with an acute onset, such as acute myocardial infarction, other acute ischemic heart diseases, acute pericarditis, acute and subacute endocarditis, acute myocarditis, cardiac arrest and heart failure, were excluded if first identified from the birth admission records. This is because the identified diseases may be the complications of pregnancy and it is only appropriate to include diseases that are present before the birth admission. We used maternal hypertension information that was recorded in either the birth data or the hospital data at birth to improve ascertainment of this condition [19].

**Table 1:** ICD10-AM diagnose and procedure codes for the eight chronic diseases.

Diseases	ICD10-AM codes
Cardiac diseases	Diagnoses: I00-02, I05-09, I10-I15, I20-25, I26-28, I30-52, O90.3, Q20-Q25 (when assessing the birth admission records codes such as I21, I24, I30, I33, I40, I46 and I50 were excluded) Procedure: 38603-00, 38600-00, 38256-00, 38256-01, 38256-02, 38278-00, 38278-01, 38284-00, 90202-00, 38470-00, 38473-00, 38281-01, 38281-02, 38281-03, 38281-04, 38281-05, 38281-06, 38281-07, 38281-07, 38281-08, 38281-09, 38281-10, 38281-00, 38278-02, 38456-07, 90203-00, 38284-01, 90219-00, 38281-11, 38281-12, 38212-00, 38209-00, 38200-00, 38203-00, 38206-00, 35324-00, 35315-00, 35315-01, 35304-01, 35305-00, 35304-00, 35305-01, 35310-00, 35310-01, 35310-03, 35310-04, 35310-02, 35310-05 (assessed only on hospital records before the birth admission)
Chronic kidney disease	Diagnoses: E10.2, E11.2, E12.2, E13.2, E14.2, I12-13, I15.0, I15.1, N00-08, N11-12, N14-16, N18-19, N25-28, N39.1, N39.2, Q60-63, T82.4, T86.1, Z49, Z94.0, Z99.2 Procedure: 36561-00, 36503-00, 36503-01, 13100-06, 13100-07, 13100-08, 13100-00 (assessed only on hospital records before the birth admission)
Asthma/Chronic obstructive pulmonary disease	Diagnoses: J40-J47, J98, R05
Psychiatric disorders	Diagnoses: F20-F25, F28-F34, F38, F39, F53.1
Pre-existing hypertension	Diagnoses: O10, O11, I10-I15 or chronic hypertension* in birth data
Pre-existing diabetes	Diagnoses: O24.0-O24.3, E10-E14 (excluded records that were also coded with O24.4)
Thyroid disorders	Diagnoses: E00-E07, E89.0, O90.5 Procedure: 30075-03, 30094-10, 30296-00, 30297-00, 30297-01, 30306-00, 30308-00, 30309-00, 30310-00, 36503-01, 90041-00, 90046-00, 90046-01, 90047-00, 90047-01, 90047-02 (assessed only on hospital records before the birth admission)
Autoimmune diseases^	Diagnoses: K50, K51, M32, L93, D69.3, G35, L40, E06.3, M05, M06, K90.0, I77.6, I80, L95, M30, M31, D51.0, G70.0, K75.4, M08.1, M45, M33.2, K74.3, M33.0, M33.1
Obstetric haemorrhage^&^	Diagnoses: O72, O67, O46.0, O44.1, O43.2, Z51.3, D62 Procedure: 13706-01, 13706-02, 13706-03, 92061-00, 92062-00, 90482-00, 90483-00

*Chronic hypertension tickbox (recorded in the NSW Midwives Data Collection): ^ Include Crohn's disease, ulcerative colitis, lupus, idiopathic, thrombocytopenic purpura, multiple sclerosis, psoriasis,

In this study, obstetric haemorrhage (refer to as 'haemorrhage') was identified from maternal hospital records for the birth admission and any associated transfer to another hospital prior to discharge home. A case of haemorrhage was determined if a record had any diagnosis code for postpartum haemorrhage (O72), intrapartum haemorrhage (O67), placenta previa with haemorrhage (O44.1), antepartum haemorrhage (O46.0), morbidly adherent placenta (O43.2), transfusion (Z51.3) or acute post-haemorrhage anaemia (D62); any procedure code for transfusions (13706-01,13706-02,13706-03,92061-00 or 92062-00) or in case of vaginal birth any procedure code for manual removal of placenta (90482-00 or 90483-00).

### Data analysis

The proportion of women with the selected chronic disease was calculated for different lengths of lookback, with the longer lookback periods including all conditions reported in the shorter periods: 'Birth' - at birth admission (day 0), 'Pregnancy' - from day 0 back to the estimated 1^st ^day of pregnancy, '2 years' - from day 0 back to 2 years, '3 years' - from day 0 back to 3 years, '4 years' - from day 0 back to 4 years and '5 years' - from day 0 back to 5 years. The first day of pregnancy was estimated by baby's date of birth minus 7 × gestation age (ranged from 18 to 44 weeks) that was recorded in the birth record. Potential risk factors for obstetric haemorrhage such as type of hospital, baby's gender, birth weight, multiple birth, gestational age, maternal age and combination of onset of labour and mode of delivery were obtained from the birth record where they are reliably reported [20].

Logistic regression was employed to determine the effect size (odds ratio) of a potential risk factor on haemorrhage after adjusting for maternal age. In the selection of independent risk factors, age was always retained in the model and a backwards elimination approach was used to progressively remove the least significant term until all terms remaining were significant (*P *< 0.05, two-sided). The capacity of a model to predict haemorrhage was evaluated using the area under the receiver-operating characteristic (ROC) curve (C-statistic), with values of 1.0 representing perfect ability and 0.5 indicating no better ability than chance. For comparing correlated C-statistics, we used *%roc *SAS^® ^macro [21] (a nonparametric approach based on generalized U-Statistics [22]).

## Results

Of 55,002 women with a first birth from 1 July 2005 through 31 December 2006, 53,438 (97.2%) linked to a birth admission record and were included in the study (Figure 1). A total of 111,647 hospital records including admissions in the five years prior to (n = 58,209) and the birth admission records (n = 53,438) were used to ascertain chronic diseases for the 53,438 women, giving a median of two records per woman (range: 1 to 263; interquartile: 1 to 3). Of the 53,438 women with a mean age of 28.8 (SD 5.7) years, 47.9% had one record (the birth admission), 22.2% had two records, 11.2% had three records, 6.2% had four records, 3.6% had five records and 9.0% had six or more records. Table 2 presents numbers of women and hospital records for each ascertainment period. In addition to the birth admissions, 1,831 women were transferred to another hospital prior to discharge from the hospital system, and both the birth and record subsequent to transfer were used for identifying haemorrhage (Figure 1). From these 55,269 records, 5,047 (9.4%) women were determined to have haemorrhage according to the definition of this study.

**Table 2:** Numbers of women and hospital records for each ascertainment period

Time sequence	No. (%) of women (N = 53,438)	No. (%) of hospital records (N = 113,478)
Transfer admission*	1,806 (3.4%)	1,831 (1.6%)
At birth admission	53,438 (100%)	53,438 (47.1%)
During pregnancy	15,284 (28.6%)	25,351 (22.3%)
Estimated day preceding pregnancy to <2 years prior to delivery	9,845 (18.4%)	16,640 (14.7%)
2 to <3 years prior to delivery	4,662 (8.7%)	6,930 (6.1%)
3 to <4 years prior to delivery	3,672 (6.9%)	5,158 (4.5%)
4 to <5 years prior to delivery	2,954 (5.5%)	4,130 (3.6%)

* Hospital records for any associated transfer to another hospital after the delivery but prior to discharge home.

The numbers of these chronic diseases ascertained from different lookback periods are presented in Table 3. In this sample, asthma/COPD was the disease with the highest prevalence (2.35%) while the thyroid disorders had the lowest prevalence (0.51%). The proportion of all cases that were ascertained from the birth admission records (=No. of cases from 'Birth'/No. of cases from '5 years') differed by disease from 17.8% for chronic renal disease to 82.0% for pre-existing diabetes. For all chronic diseases, the prevalence increased with increasing length of lookback period. However, the rate of the increase was much slower after 2 to 3 years than for the more recent periods. The additional (case) ascertainment from year 4 to year 5 (i.e. [number at year 5 - number at year 4]/number at year4) was small, ranging from 0.5% for pre-existing hypertension to 6.8% for asthma/COPD.

**Table 3:** Cumulative frequency and relative frequency of cases ascertained at different lookback periods and the prevalence of diseases for the 53,438 women by disease type

	Lookback period:					
	'Birth'	'Pregnancy'	'2 years'	'3 years'	'4 years'	'5 years'
**Cardiac diseases**						
No.	194	273	357	392	425	445
% of total cases	43.6%	61.3%	80.2%	88.1%	95.5%	100.0%
Prevalence	0.36%	0.51%	0.67%	0.73%	0.80%	0.83%
**Chronic renal disease**						
No.	67	217	279	324	354	377
% of total cases	17.8%	57.6%	74.0%	85.9%	93.9%	100.0%
Prevalence	0.13%	0.41%	0.52%	0.61%	0.66%	0.71%
Asthma/COPD*						
No.	473	682	984	1,087	1,175	1,255
% of total cases	37.7%	54.3%	78.4%	86.6%	93.6%	100.0%
Prevalence	0.89%	1.28%	1.84%	2.03%	2.20%	2.35%
**Psychiatric disorders**						
No.	325	424	619	705	783	829
% of total cases	39.2%	51.1%	74.7%	85.0%	94.5%	100.0%
Prevalence	0.61%	0.79%	1.16%	1.32%	1.47%	1.55%
**Pre-existing hypertension**						
No.	420	515	535	540	546	549
% of total cases	76.5%	93.8%	97.4%	98.4%	99.5%	100.0%
Prevalence	0.79%	0.96%	1.00%	1.01%	1.02%	1.03%
**Pre-existing diabetes**						
No.	250	265	289	297	300	305
% of total cases	82.0%	86.9%	94.8%	97.4%	98.4%	100.0%
Prevalence	0.47%	0.50%	0.54%	0.56%	0.56%	0.57%
**Thyroid disorders**						
No.	173	204	240	253	266	275
% of total cases	62.9%	74.2%	87.3%	92.0%	96.7%	100.0%
Prevalence	0.32%	0.38%	0.45%	0.47%	0.50%	0.51%
**Autoimmune diseases^**						
No.	202	247	323	374	401	426
% of total cases	47.4%	58.0%	75.8%	87.8%	94.1%	100.0%
Prevalence	0.38%	0.46%	0.60%	0.70%	0.75%	0.80%

* Chronic obstructive pulmonary disease

^ Include Crohn's disease, ulcerative colitis, lupus, idiopathic, thrombocytopenic purpura, multiple sclerosis, psoriasis, autoimmune thyroiditis, rheumatoid diseases, Coeliac disease, vasculitis, pernicious anemia, myasthenia gravis, autoimmune hepatitis, ankylosing spondylitis, polymyositis and primary biliary cirrhosis

Based on birth admission records only and after adjusting for maternal age, significant risk factors for haemorrhage were cardiac disease (OR 1.55, 95% CI: 1.03 to 2.33; *P *= 0.04), chronic renal disease (OR 3.00, 95% CI: 1.71 to 5.26; *P *< 0.001) and psychiatric disorders (OR 1.67, 95% CI: 1.22 to 2.27; *P *= 0.001) (Table 4). However, cardiac disease was not statistically significant when we included cases ascertained from hospital records that were more than two years prior to delivery. For these three diseases, the effect size decreased consistently with increasing length of lookback period. For the other five diseases presented in Table 3, there was only a small change in the effect size from one lookback period to another despite the identification of more women with the condition. For example, for pre-existing diabetes age-adjusted OR based on lookback period of 'Birth', 'Pregnancy', '2 years', '3 years', '4 years' or '5 years' was 1.06, 1.12, 1.10, 1.15, 1.14 and 1.12 respectively; and for pre-existing hypertension was 1.22, 1.18, 1.17, 1.16, 1.15 and 1.14 respectively (Table 4).

**Table 4:** Age-adjusted^&^odds ratio (OR) for potential predictors of obstetric haemorrhage for different lookback periods

Type of diseases	Lookback periods:					
	'Birth'	'Pregnancy'	'2 years'	'3 years'	'4 years'	'5 years'
**Cardiac diseases**						
OR (95% CI)	1.55 (1.03-2.33)	1.45 (1.02-2.07)	1.42 (1.04-1.93)	1.34 (0.99-1.81)	1.28 (0.95-1.72)	1.21 (0.90-1.63)
P value	0.04	0.04	0.03	0.06	0.11	0.21
**Chronic renal disease**						
OR (95% CI)	3.00 (1.71-5.26)	1.68 (1.16-2.45)	1.58 (1.13-2.22)	1.49 (1.08-2.06)	1.45 (1.07-1.99)	1.42 (1.05-1.92)
P value	<0.001	0.007	0.008	0.02	0.02	0.02
**Psychiatric disorders**						
OR (95% CI)	1.67 (1.22-2.27)	1.50 (1.13-1.98)	1.36 (1.07-1.73)	1.30 (1.03-1.63)	1.28 (1.02-1.59)	1.24 (1.00-1.54)
P value	0.001	0.005	0.01	0.03	0.03	0.05
**Pre-existing hypertension**						
OR (95% CI)	1.22 (0.90-1.65)	1.18 (0.89-1.55)	1.17 (0.89-1.54)	1.16 (0.88-1.52)	1.15 (0.87-1.51)	1.14 (0.87-1.50)
P value	0.20	0.25	0.25	0.28	0.32	0.35
**Pre-existing diabetes**						
OR (95% CI)	1.06 (0.70-1.60)	1.12 (0.76-1.66)	1.10 (0.76-1.61)	1.15 (0.80-1.66)	1.14 (0.79-1.64)	1.12 (0.77-1.61)
P value	0.79	0.52	0.61	0.46	0.49	0.56
Asthma/COPD*						
OR (95% CI)	1.22 (0.92-1.63)	1.20 (0.94-1.53)	1.19 (0.98-1.46)	1.16 (0.95-1.41)	1.13 (0.94-1.37)	1.09 (0.90-1.31)
P value	0.18	0.15	0.09	0.14	0.20	0.37
**Thyroid disorders**						
OR (95% CI)	1.38 (0.88-2.16)	1.39 (0.92-2.10)	1.41 (0.97-2.06)	1.38 (0.95-2.00)	1.39 (0.97-2.00)	1.34 (0.93-1.92)
P value	0.16	0.12	0.07	0.09	0.07	0.11
**Autoimmune diseases^**						
OR (95% CI)	1.34 (0.88-2.04)	1.31 (0.89-1.92)	1.23 (0.87-1.73)	1.17 (0.84-1.62)	1.23 (0.90-1.68)	1.24 (0.92-1.67)
P value	0.17	0.17	0.24	0.35	0.19	0.17

& Age was divided into three groups: 15-19, 20-34 and ≥35 and treated as a categorical variable in the analyses.

* Chronic obstructive pulmonary disease

^ Include Crohn's disease, ulcerative colitis, lupus, idiopathic, thrombocytopenic purpura, multiple sclerosis, psoriasis, autoimmune thyroiditis, rheumatoid diseases, Coeliac disease, vasculitis, pernicious anemia, myasthenia gravis, autoimmune hepatitis, ankylosing spondylitis, polymyositis and primary biliary cirrhosis

In multivariate analysis, only chronic renal disease (adjusted OR 2.85, 95% CI: 1.58 to 5.12; *P *< 0.001) and psychiatric disorders (adjusted OR 1.48, 95% CI: 1.08 to 2.02; *P *= 0.01) were independent risk factors for haemorrhage using the birth admission records. These results were adjusted for type of hospital, baby's gender, birth weight, multiple birth, gestational age, maternal age and combination of onset of labor and mode of delivery (Table 5). This model has a C-statistic of 0.624. The predictive ability did not improve with any extension of the lookback period for ascertaining the two diseases (all *P *> 0.29), and the C-statistic remained about the same (0.624) for models using hospital records from lookback periods of 'Pregnancy', '2 years', '3 years', '4 years' and '5 years'. This was also the case when comparing to a model that included two variables (i.e. one for the birth admission and the other for the lookback period) for each of the two chronic diseases (C-statistic 0.624 vs 0.624, *P *= 0.95). Both additional variables were not statistically significant in the model (both *P *> 0.71).

**Table 5:** Independent risk factors of obstetric haemorrhage and C-statistics

	Model 1* (n = 53,191):		Model 2**(n = 53,191):	
**Covariates:**	**OR (95%CI)**	**P**	**OR (95%CI)**	**P**
				
**Chronic renal diseases **(yes)	2.85 (1.58-5.12)	<0.001	1.63 (1.11-2.40)	0.01
**Psychiatric disorders **(yes)	1.48 (1.08-2.02)	0.01	1.38 (1.04-1.84)	0.03
**Baby's gender **(female)	1.15 (1.09-1.22)	<0.001	1.15 (1.09-1.22)	<0.001
**Birth weight **(per 100 g)	1.05 (1.05-1.06)	<0.001	1.05 (1.05-1.06)	<0.001
**Multiple births **(yes)	2.28 (1.86-2.78)	<0.001	2.27 (1.86-2.78)	<0.001
**Gestational age**		<0.001		<0.001
Preterm (20 - 36 weeks)	2.11 (1.85-2.40)		2.12 (1.86-2.41)	
Term (37 - 41 weeks)	1.00		1.00	
Postterm (≥42 weeks)	0.99 (0.82-1.19)		0.99 (0.82-1.20)	
**Maternal age**		0.11		0.10
<20 years	1.03 (0.92-1.15)		1.02 (0.91-1.15)	
20 - 34 years	1.00		1.00	
≥35 years	1.10 (1.01-1.19)		1.10 (1.01-1.19)	
**Onset of labor + mode of delivery**		<0.001		<0.001
Elective CS^ (no labor)	1.07 (0.96-1.20)		1.08 (0.96-1.20)	
Emergency CS + induction/augmentation	0.87 (0.78-0.98)		0.88 (0.78-0.98)	
Instrumental + induction/augmentation	1.99 (1.81-2.19)		1.99 (1.82-2.19)	
Vaginal + induction/augmentation	1.43 (1.31-1.56)		1.43 (1.31-1.56)	
Emergency CS (spontaneous labor)	0.77 (0.67-0.90)		0.77 (0.67-0.90)	
Instrumental (spontaneous labor)	1.58 (1.40-1.77)		1.58 (1.41-1.77)	
Vaginal (spontaneous labor)	1.00		1.00	
**Type of hospital (**tertiary vs. other)	1.54 (1.45-1.64)	<0.001	1.54 (1.45-1.64)	<0.001
				
	**C-statistic **(95% CI)		**C-statistic **(95% CI)	
				
**Predictive ability**	0.6240 (0.6157-0.6322)		0.6238 (0.6156-0.6320)	
**P for comparing the two correlated C statistics: **0.61				

* Using 'Birth' records

** Using 'Pregnancy' records

^ Cesarean section

## Discussion

This study showed that longer ascertainment periods resulted in improved identification of chronic disease history among pregnant women. Surprisingly, extension of the lookback period up to five years for chronic diseases did not increase the estimated risk effect of any predictions of haemorrhage, and contributed little to the performance of the haemorrhage predictive model. These results indicate that the effort of accessing previous hospital records for the completeness of comorbidity information is not always worthwhile.

As anticipated, the ascertainment rate of a chronic disease in this and other studies [6] increased progressively with increasing length of the lookback period. We hoped that a five-year ascertainment period for a chronic disease would give good estimation of the population prevalence in the study of young and generally healthy women. In this study, the population prevalence of chronic renal disease in young women in NSW was estimated to be around 0.7% based on a five-year ascertainment period. This is within the range of internationally reported prevalence (0.5 to 1.3%) [23-26]. The rate of 0.8% for cardiac diseases (mainly congenital heart disease in this population) in this study also appears to provide a good estimate of the population prevalence. Congenital heart disease occurs in approximately 1% of newborn babies worldwide [27] and about 80% of patients with such disease survive to adulthood [28]. The prevalence of 0.6% for pre-existing diabetes in this study is similar to the population prevalence of 0.7% in Australian women aged <45 years, 2004 to 2005 [29]. The rate of 0.51% for thyroid disorders in this study is similar to the estimated rate of clinical hypothyroidism or hyperthyroidism in the USA (0.43%), although the majority of thyroid disease is subclinical [30,31].

However, our study indicated that the prevalence of some diseases (i.e. asthma and chronic hypertension) was under-estimated. This is likely to be related to the fact that hospital data only identifies diseases/conditions that require hospitalisation or that affect a hospital admission. Although lookback over 5 years increased the identification of asthma from 0.9% to 2.4%, this still represents poor identification of women with asthma. The National Health Survey 2004-05 reported that 13.5% of Australian women aged 15 to 45 years had asthma and 3% of the population had COPD (emphysema and/or bronchitis) [32]. Similarly a validation study of 1184 pregnant women in NSW reported the prevalence of asthma to be 12% in pregnancy and the sensitivity of the recording of asthma as a comorbidity during maternal birth admissions was only 12.3% [33]. The prevalence of chronic hypertension (1% with ≥2 years of lookback) is lower than the prevalence of antihypertensive drug use in 25 to 34 year olds in NSW in 1999 (1.4%), but 26.3% of pregnant women were < 25 years in our population [34]. Other limitations of using longitudinally linked hospital records included missing ascertainment periods (e.g. migration or admission to hospitals outside NSW) and outpatient data, the assumption of disease chronicity and changes in diagnostic criteria for a disease over time.

With regard to predictive ability, information from prior hospital admissions might not improve the capacity of a predictive model if it were simply used to increase the number of cases with a condition. In a study of 61,815 patients, Kim and Ahn [5] reported no significant improvement in the predictive capacity of in-hospital mortality of a model with 3-years inpatient comorbidity score (either Elixhauser or Charlson) compared to a model with 1-year inpatient comorbidity score. Zhang et al. [3] also reported that models for 1-year mortality prediction among elderly patients using 1-year inpatient Charlson score or 2-years inpatient Charlson score were almost identical. Extra cases identified from prior admissions might be less severe or at an early stage of the illness but are treated equally to the cases from the index admission in the analysis. This might explain the finding of no improvement in the statistical performance by this and other studies.

On the other hand, Zhang et al. [3] found increased predictive capacity if comorbidity information from year 1 and year 2 inpatient records for the Charlson score were entered separately into the model. Preen et al. [6] reported similar findings, and found that C-statistics for 1-year mortality prediction in medical patients and procedural patients were 0.892 and 0.917 respectively for a model with a comorbidity score of the index admission and increased to 0.900 and 0.923 respectively for a model with two comorbidity scores: one for the index admission and another for 5-year prior admissions. In another study of the contribution to model performance in predicting in-hospital mortality made by extra information from a 3-year lookback period, Stukenborg et al. [8] reported that comorbidity risk adjustment (either Deyo/Charlson or Elixhauser method) achieved the best performance in various groups of hospital patients when comorbidity information from the index and prior admissions were treated as separate covariates in a model. Nevertheless, they also concluded that ascertaining information from prior admissions provided little improvement in the explanatory power of risk adjustment methods. Using information from the index and prior admissions as independent indicators might allow the model to distinguish late-stage from early-stage cases because more severe cases were more likely to be ascertained more than once and thus produce some improvement in the statistical performance.

With regard to effect estimation, increasing the number of cases with a disease/condition by using information from prior hospital admissions could produce an effect size smaller than that estimated using only the index record (i.e. only severe or active cases). In this study we found that the more remote (in time) that hospitalisations with chronic disease were reported, the smaller the effect the disease had on haemorrhage. One explanation for this could be that conditions that were ascertained from previous hospital records might have been treated and well controlled or be less severe than conditions identified from the index records. The effect of a risk factor on a particular outcome is likely to be dependent not only on the risk factor but also its severity, and a more severe instance is more likely to be ascertained in recent records than in older records [35]. Not much additional comorbidity information had been gained in this generally young and healthy population using a longer lookback period. Thus, this study indicates that the findings of lookback studies may not be generalisable between young and older populations.

Chronic renal disease (via anemia) and psychiatric disorders (via medication) may place women at increased risk of obstetric haemorrhage [36]. Pregnant women with chronic renal disease or treated psychiatric disorders which complicate the pregnancy or are associated with hospitalisation during the pregnancy should be considered to be at risk of haemorrhage and be treated accordingly.

## Conclusions

A five-year ascertainment period for a chronic disease improves estimation of the population prevalence in a young and generally healthy population if the disease required treatment in hospital. On the other hand, diseases that do not require hospitalisation or cases with no obvious symptoms or in subclinical categories would usually not be picked up using longitudinally linked hospital records. In the case of haemorrhage prediction, comorbidity information from prior hospital admissions did little to improve the haemorrhage modelling. For estimating the effect size of a risk factor, the most appropriate lookback period should be determined by the study objective.

## Competing interests

The authors declare that they have no competing interests.

## Authors' contributions

JSC performed the statistical analysis and drafted the manuscript. CLR and JBF conceived of the study, participated in its design and revised it critically for important intellectual content. JMS contributed to the study design, statistical analysis and interpretation of the results. All authors read and approved the final manuscript.

## Pre-publication history

The pre-publication history for this paper can be accessed here:

http://www.biomedcentral.com/1471-2288/11/68/prepub
